# Temporal and spatial variation in population structure among brooding sea stars in the genus *Leptasterias*

**DOI:** 10.1002/ece3.7283

**Published:** 2021-03-04

**Authors:** Laura M. Melroy, C. Sarah Cohen

**Affiliations:** ^1^ Department of Biology Estuary & Ocean Science Center San Francisco State University Tiburon CA USA

**Keywords:** echinoderm, ecological genetics, low dispersal, phylogeography, species complex, temporal structure

## Abstract

Temporal genetic studies of low‐dispersing organisms are rare. Marine invertebrates lacking a planktonic larval stage are expected to have lower dispersal, low gene flow, and a higher potential for local adaptation than organisms with planktonic dispersal. *Leptasterias* is a genus of brooding sea stars containing several cryptic species complexes. Population genetic methods were used to resolve patterns of fine‐scale population structure in central California *Leptasterias* species using three loci from nuclear and mitochondrial genomes. Historic samples (collected between 1897 and 1998) were compared to contemporary samples (collected between 2008 and 2014) to delineate changes in species distributions in space and time. Phylogenetic analysis of contemporary samples confirmed the presence of a bay‐localized clade and revealed the presence of an additional bay‐localized and previously undescribed clade of *Leptasterias*. Analysis of contemporary and historic samples indicates two clades are experiencing a constriction in their southern range limit and suggests a decrease in clade‐specific abundance at sites at which they were once prevalent. Historic sampling revealed a dramatically different distribution of diversity along the California coastline compared to contemporary sampling and illustrates the importance of temporal genetic sampling in phylogeographic studies. These samples were collected prior to significant impacts of Sea Star Wasting Disease (SSWD) and represent an in‐depth analysis of genetic structure over 117 years prior to the SSWD‐associated mass die‐off of *Leptasterias*.

## Graphical abstract

*Leptasterias* is a genus of brooding sea stars with low dispersal and restricted gene flow. Genetic analysis of *Leptasterias* species along the California coastline revealed high temporal variability and genetic structure over 117 years Photo by Jeyna Perez.

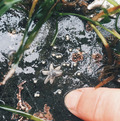

## 1 INTRODUCTION

The marine environment is heterogeneous with many instances of cryptic speciation and genetic differentiation due to adaptive and neutral processes of divergence (Bohonak, 1999; Boissin et al., 2008). Geographic patterns of genetic variation in the marine environment are shaped by life history (Cahill et al., 2017), oceanographic and transport processes (Kelly & Palumbi, 2010; Perrin et al., 2004), sea level, and land changes (Hellberg et al., 2001), and selection (Koehn, 1978; Puritz & Toonen, 2011). These processes influence gene flow and population structure of marine invertebrates on both temporal and spatial scales (Fenderson et al., 2020; Je Lee & Boulding, 2009).

In comparison with marine organisms with planktonic larvae, marine direct‐developers and brooders tend to have lower vagility, lower levels of gene flow and greater genetic structure among populations, a higher potential for local adaptation, and more frequent speciation and extinction events (Barbosa et al., 2013; Jablonski, 1986; Keever et al., 2013; Kelly & Palumbi, 2010; Levin, 2006; Strathmann & Strathmann, 1982). However, predictions of genetic structure based on developmental mode are not always consistent (Becker et al., 2007; Bradbury et al., 2008; Sotka et al., 2004). These inconsistencies have been explained by intrinsic and extrinsic factors influencing dispersal such as larval behavior, currents and circulation, habitat discontinuity, and geographic features (Avise, 1992; Billot et al., 2003; Kamel et al., 2014; Selkoe et al., 2010; Winston, 2012).

While studies of low‐dispersers commonly find high spatial genetic structure among populations (Collin, 2001; Hellberg, 1996; Hunt, 1993), studies assessing temporal genetic structure among organisms with low‐dispersal life histories are less common. Je Lee and Boulding (2009) compared the genetic structure of four littorinid gastropods, two of which were planktonic‐dispersers and two of which were brooders, over 10 years and four sites leading to predictions of variability based upon inferred dispersal ability. On a temporal scale, planktonic‐dispersers are predicted to have high levels of genetic turnover between generations due to stochastic processes affecting larval mortality and high gene flow between populations (Eldon et al., 2016; Je Lee & Boulding, 2009). According to the sweepstakes hypothesis, relatively few individuals will contribute to the recruits of the next generation due to high variance in success at varied life‐history stages, leading to high temporal genetic variability (Hedgecock, 1994; Johnson & Black, 1982). Conversely, low‐dispersers with significant parental care are expected to experience low juvenile mortality and have low levels of temporal variability across generations (Je Lee & Boulding, 2009; Puritz et al., 2017). In brooders, low gene flow from other populations also contributes to high population genetic stability over time. Je Lee and Boulding (2009) provide predictions of limited temporal genetic structure for low‐dispersers over 10 years. Additional studies are needed to test this pattern in other taxa over variable lengths of time. Assessing temporal genetic stability in brooding species will provide insight into long‐term environmental and demographic processes contributing to population structure and inform population responses to large‐scale environmental changes.

*Leptasterias* is a genus of small‐bodied lecithotrophic sea stars ranging from Alaska to central California composed of several cryptic species complexes. *Leptasterias* occur in rocky intertidal and subtidal habitats, typically measure less than 6 cm from ray tip to ray tip (Chia, 1966; Fisher, 1930; Niesen, 1973), and mature around 2 years of age (Menge, 1974). *Leptasterias* are lecithotrophic and females brood their young underneath their rays until the fully developed juveniles crawl away to disperse (Barreto & Bauer, 2019; Chia, 1966; Menge, 1975). Due to their brooding life history and small size, these sea stars have limited dispersal to new sites. Dispersal likely occurs by individuals rafting on macroalgae or other floating substrate; long‐distance dispersal is possible though likely infrequent (Highsmith, 1985; Parker & Tunnicliffe, 1994).

Due to the limited vagility of these sea stars and high susceptibility to local selection pressures like algal blooms and disease outbreaks, *Leptasterias* can be a coastal‐indicator species reflecting local environmental health; however, proper species identification is necessary to assess changing distributions, abundances, and population health. Assessing cryptic diversity is also important for providing baseline data for monitoring ecological effects of mortality events and other environmental perturbations, especially as these types of events are predicted to increase with global climate change (Harvell et al., 2004; Jurgens et al., 2015). For example, Sea Star Wasting Disease (SSWD) is a syndrome that resulted in mass mortalities in many Pacific Coast sea star populations and is multifactorial in cause (Bates et al., 2009; Eisenlord et al., 2016; Hewson et al., 2014, 2018; Kohl et al., 2016; Menge et al., 2016). SSWD impacts on sea star genera, including *Pisaster* and *Pycnopodia*, were first noted in central California in 2013 (Eisenlord et al., 2016). Collections for this study were completed in 2014, before major impacts of SSWD were evident in *Leptasterias* (Eberl et al., 2017; Eisenlord et al., 2016; Jaffe et al., 2019; MARINe, 2015).

Many lineages within the *Leptasterias* genus have an unresolved taxonomic status. In several broad‐scale analyses of the *Leptasterias* genus, cryptic lineages were identified within both *L. hexactis* and *L. aequalis* using mitochondrial molecular data (Foltz et al., 2008; Hrincevich et al., 2000). *Leptasterias hexactis* is comprised of two distinct clades: *L. hexactis* C found in Washington and *L. hexactis* G found in Alaska. *Leptasterias aequalis* is comprised of four allopatric and sympatric clades: *L. aequalis* B in Washington, *L. aequalis* A ranging from Washington to north of San Francisco Bay, *L. aequalis* D ranging from Washington to south of Monterey Bay, and *L. aequalis* K ranging from Cape Mendocino to south of Monterey Bay. Morphological characters are challenging for identification within the *Leptasterias* genus due to high morphological variability within and among clades (Foltz et al., 1996) and potential hybridization (Foltz, 1997). Taxonomic uncertainty exists for *L. aequalis* D, which is also referred to as *Leptasterias pusilla* in some literature (Foltz et al., 2008). Fine‐scale genetic analysis of *Leptasterias* will contribute to taxonomic revision and resolution in this genus.

Past studies on the broad‐scale distributions of *Leptasterias* spp. do not account for the fine‐scale cryptic diversity within the genus and previous studies addressed the need for fine‐scale analysis (Foltz, Nguyen, Nguyen, & Kiger, 2007, 2008). Indeed, a recent study used one mitochondrial locus to reveal the presence of a previously undescribed clade, Clade Y, localized around the San Francisco Bay outflow (Melroy et al., 2017). Here, we investigate temporal and spatial population structure using nuclear and mitochondrial sequence data with widespread contemporary sampling and historic museum sampling. Previous range estimates of *Leptasterias* might be incorrect as museum samples were historically identified based on unreliable morphological characters; molecular identification in this study could contradict early classifications. Multilocus sequence data will be used to (a) clarify the phylogenetic relationship of California *Leptasterias* lineages, and (b) assess temporal and spatial patterns of population structure. We predict high population structure and high temporal stability in structure over time due to the low‐dispersal potential of *Leptasterias*.

## 2 METHODS

### 2.1 Sample collection and DNA extraction

Three hundred forty‐five adult *Leptasterias* individuals were collected from 17 intertidal sites on the Pacific Coast between December 2008 and July 2014 (Table 1, DFW Scientific Collecting Permit SC‐12882). One ray was collected from each individual and individuals were collected if there was at least one meter of separation to avoid family groups. Ray tissue was stored in 95% ethanol. Samples are stored at San Francisco State University. Alaskan samples were provided courtesy of Marnie Chapman, Sara Caldwell, and Sherry Tamone from the University of Alaska Southeast. Several tube feet from each ray sample were used for DNA extraction.

**TABLE 1:** Summary data for each sampling locality and year sampled

Location	Site	Lat, Long	Year	Region	COI		D‐Loop		i51	
	Code				N	Nh (Pr)	N	Nh (Pr)	N	Na (Pr)
*Contemporary Sites*					*305*	*73 (34)*	*322*	*59 (45)*	*289*	*46 (27)*
Auke Bay, AK	AB	58.38, −134.64	2014	Alaska	5	2 (1)	5	3 (1)	5	2 (1)
Sage Bay, AK	SB	57.05, −135.34	2014	Alaska	5	1	5	2	5	5 (1)
Griffin Bay, WA	GB	48.50, −123.02	2014	Washington	19	4 (4)	19	4 (4)	17	6 (2)
Twin Cove, CA	TC	38.43, −123.12	2011	Northern	20	10 (6)	20	7 (5)	20	9 (2)
Marshall Gulch, CA	MG	38.37, −123.07	2008	Northern	14	6 (4)	14	5 (3)	6	6
Bodega Bay, CA	BB	38.30, −123.06	2008	Northern	8	3	7	4 (2)	8	9 (1)
Duxbury Reef, CA	DR	37.89, −122.70	2014	Bay‐proximal	16	2 (2)	16	2 (1)	16	4
Slide Ranch, CA	SR	37.87, −122.60	2014	Bay‐proximal	21	3 (2)	21	4 (3)	21	4
Muir Beach, CA	MB	37.86, −122.59	2013	Bay‐proximal	27	4 (1)	44	7 (4)	25	9 (4)
Rodeo Beach, CA	RB	37.82, −122.53	2014	Bay‐proximal	31	4 (2)	31	4 (1)	31	7 (2)
Point Bonita, CA	PB	37.81, −122.53	2014	Bay‐proximal	20	4	20	6 (2)	20	8 (2)
Lands End, CA	LE	37.78, −122.50	2013	Bay‐proximal	20	1	20	2 (1)	20	6 (3)
Mussel Rock, CA	MR	37.50, −122.49	2008	Bay‐proximal	20	2 (1)	20	1	19	4
Half Moon Bay, CA	HMB	37.18, −122.39	2011–14	Southern	7	4 (3)	8	5 (4)	8	9 (2)
Pigeon Point, CA	PP	37.18, −122.39	2013–14	Southern	32	9 (1)	33	14 (8)	32	12 (3)
Point Pinos, CA	PN	36.64, −121.95	2014	Southern	20	6 (2)	20	7 (3)	20	9 (4)
Carmel Point, CA	CP	36.54, −121.93	2014	Southern	20	8 (5)	20	5 (3)	16	5
*Historic Sites*	*CAS ID*				*61*	*73, (7)*				
Crescent City, CA	CC	201227	1897		6	2 (1)				
Pacific Grove, CA	PG	191756	1897		9	2				
Pacific Grove, CA	PG	108854	1909		6	1				
San Simeon, CA	SS	115491	1916		2	2				
Bodega Head, CA	BB	115521	1963		3	1				
Pigeon Point, CA	PP	7676	1971		3	2				
Pigeon Point, CA	PP	191755, 7642	1972		4	3 (1)				
Franklin Point, CA	FP	7645	1972		1	1				
Point Bonita, CA	PB	115524	1973		2	1				
Diablo Canyon, CA	DC	135003	1974		1	1				
SE Farallon Islands, CA	FI	4826	1977		3	2 (1)				
Piedras Blancas, CA	PE	164031, 115497	1978		4	4				
Duxbury Reef, CA	DR	115493	1998		1	1				
Lonesome Cove, WA	LC	DF	1998		2	2				
Pigeon Point, CA	PP	DF	1998		8	8 (4)				
Franklin Point, CA	FP	DF	1998		2	2 (1)				

Note

Estimated latitude and longitude coordinates for collection sites (Lat, Long), number of samples successfully sequenced for each locus (N), number of haplotypes found per site (Nh), number of alleles found at each site (Na), number of singletons per site (Pr) are provided for each collection site and time point. Region indicates grouping used for AMOVA analysis. Site code indicates population abbreviations found in figures. CAS ID is the specimen identification number for the California Academy of Sciences Invertebrate Zoology Collection and DF indicates samples from David Foltz (Louisiana State University). Year indicates the collection date.

Historic samples of *Leptasterias* spp. collected between 1897 and 1998 were obtained from the Invertebrate Zoology collection at the California Academy of Sciences or gifted from David W. Foltz (Louisiana State University). Whole stars were collected on the Pacific coast ranging from Lonesome Cove, Washington to Diablo Canyon, California (Table 1). Several tube feet from each sea star were transferred from ethanol into milli‐Q water and left on a shaker for 2 days to remove excess ethanol prior to extraction. Sampling was meant to be non‐destructive with minimal tissue removal and whole stars were placed back into the invertebrate collection upon tube feet removal. All DNA extractions were carried out using NucleoSpin Tissue Columns (Macherey‐Nagel Inc), except for samples collected in 2008 from Marshall Gulch, Bodega Bay, and Mussel Rock, which were extracted with a phenol‐chloroform extraction.

### 2.2 Control region amplification

Forward primer E16Sa (Smith et al., 1993) and reverse primer Star‐L (Flowers & Foltz, 2001) were used for amplification of 286 bp of the putative control region and 8 bp of the conserved 3’ end of the large ribosomal subunit 16S gene (henceforth referred to in entirety as D‐Loop for simplicity). PCR reactions for contemporary samples had the following components: 5–500 ng DNA template, 0.2 µM of each primer, 1X PE II Buffer, 1 mM dNTPs, 2.5 mM MgCl₂, 1.25 µg BSA, 1 unit of Taq DNA polymerase (New England Biolabs, NEB) and milli‐Q water up to 25 uL final volume. Thermal cycling conditions were: initial denaturation at 94°C for 120 s, 30 cycles of denaturation at 94°C for 30 s, annealing at 45°C for 60 s, extension at 72°C for 60 s, and a final extension at 72°C for 300 s. If amplification in historic samples was not successful after 30 cycles, a new reaction was amplified using 35 cycles.

### 2.3 COI amplification

Primers were designed for this study from published mitochondrial cytochrome oxidase subunit I (COI) sequences of *L. aequalis* and *L. hexactis* (Foltz et al., 2008; Hrincevich et al., 2000). Forward primer COILF, 5′ GCA‐GGA‐TTT‐ACC‐CAC‐TGA‐TTT‐C 3′ and reverse primer COILR, 5′CCT‐GGC‐TTC‐ACA‐GGC‐AGA‐T 3′ amplified 378 bp of COI, 68 bp of tRNA‐Arg, and 90 bp of ND4L genes (henceforth referred to as COI for simplicity). PCR reactions were carried out using the same reaction concentrations and volumes as in D‐Loop amplification. Thermal cycling conditions were: initial denaturation at 96°C for 120 s, 35 cycles of denaturation at 94°C for 30 s, annealing at 46°C for 30 s, extension at 72°C for 60 s, and a final extension at 72°C for 300 s. For all historic samples, thermocycling conditions were run for 40 cycles.

### 2.4 Intron amplification

Five Exon Primed Intron Crossing (EPIC) loci (Chenuil et al., 2010; Gérard et al., 2013) were screened for amplification in *Leptasterias* based on successful amplification in other echinoderm taxa: i1, i9, i39, i43, and i51. One EPIC locus which offered the highest resolution among sites and clades was chosen and optimized for population genetic analyses. The i51 primer pair amplified a region in the gene group UDP‐N‐acetylglucosaminyl‐transferase (Chenuil et al., 2010). Primers were redesigned for i51 to decrease primer degeneracy. Forward primer i51LF GAT‐CGA‐CCC‐AGC‐CAC‐ATT and reverse primer i51LR TTG‐AAG‐CAA‐CAG‐GGG‐AGA‐AG were exclusively used to amplify a 277 base‐pair intronic region. PCR reactions were the same as D‐Loop and COI, but used 0.1 µM of each primer. Thermal cycling conditions were: initial denaturation at 96°C for 60 s, 35 cycles of denaturation at 94°C for 40 s, annealing at 45°C for 30 s, extension at 72°C for 40 s, and a final extension at 72°C for 120 s.

### 2.5 Sequencing reactions

Amplification of PCR templates was assessed with gel electrophoresis using a 1.5% agarose gel stained with ethidium bromide. PCR products were cleaned using a SAP/EXO reaction following manufacturer's instructions (Affymetrix). Cycle sequencing reactions were carried out in the reverse direction using the 1/8 reaction BigDyeTerminator v3.1 (Applied Biosystems, ABI). Products were sequenced using an ABI 3130 genetic analyzer. Cloning was used to resolve and confirm a subset of alleles for i51 in heterozygous individuals using Vector System II, pGEM‐T (Promega).

### 2.6 Phylogenetic analysis

COI, D‐Loop, and i51 sequences were edited by eye in Geneious v7.1.7 (Kearse et al., 2012) and aligned separately using the MUSCLE algorithm. The COI alignment was translated in Mesquite v3.0.4 (Maddison & Maddison, 2007) to ensure the correct reading frame was used and to determine base saturation and nucleotide position changes. D‐Loop and COI haplotypes were aligned to published *Leptasterias* sequences for phylogenetic analyses (Table S1). *Leptasterias camtschatica* was chosen as an outgroup based on previous phylogenetic analysis identifying it as a sister group *to L. hexactis* and *L. aequalis* (Foltz et al., 2008). Maximum likelihood (ML) analyses were performed in PAUP* v.4.0 (Swofford, 2001) for D‐Loop, COI and i51 haplotypes. Indels were treated as both missing and informative in separate analyses. The automated model selection feature was used to choose the most appropriate nucleotide substitution model using the Akaike Information Criterion (AIC; Posada & Crandall, 1998). TN93 with gamma site heterogeneity (Tamura & Nei, 1993) was used for D‐Loop and COI and HKY + G (Hasegawa et al., 1985) was used for i51 after phasing (see below). Bootstrap analyses were performed using a Jukes‐Cantor neighbor‐joining tree as the starting tree for a heuristic search with 1,000 replicates. ML analysis was performed on the full COI dataset, and then again on the dataset excluding third‐position changes.

Bayesian's analysis was performed in MrBayes v.3.2 (Huelsenbeck & Ronquist, 2001). Metropolis‐coupled Markov Chain Monte Carlo's (MCMCMC) methods were employed for all loci using the HKY + G model of nucleotide evolution. With each tree search, four parallel searches were run for 2 million generations with chains sampled every 500 generations. Trees prior to a split frequency value of 0.01 were discarded as the burn‐in. Trees were constructed for D‐Loop and COI separately, and constructed with D‐Loop and COI concatenated. The PHASE (Stephens et al., 2001) algorithm in DnaSP v5.10 (Rozas et al., 2003) was used to resolve the allelic phase for single nucleotide polymorphisms in i51 sequences. Allelic phase that could not be resolved with greater than 60% confidence were not used in the analysis (only one individual was excluded for less than 60% confidence). Only three individuals were assigned a phase with confidence less than 98%. The i51 phased haplotype alignment was imported into Seqstate v.1.4.1 (Müller, 2005) to code indels as simple characters (Simmons & Ochoterena, 2000) and complex characters (Müller, 2006). A phylogenetic tree was estimated in MrBayes for i51 using indels as both missing characters and coded as informative characters.

A log‐likelihood value was calculated to determine whether a molecular clock was appropriate for the data using BEAST v1.8.2 (Drummond et al., 2012), and the *p*‐value was non‐significant indicating the application of a molecular clock to be appropriate. Divergence times were measured between concatenated historic and contemporary mtDNA haplotypes to estimate the time of differentiation between previously undescribed clades within *L. aequalis* in BEAST. Nuclear data were omitted due to low resolution. The TN93 + G substitution model was employed in BEAST. An uncorrelated lognormal relaxed clock with an estimated substitution rate was used with all tips set to zero. *Leptasterias aequalis* was constrained as monophyletic, but taxon sets within *L. aequalis* were not constrained as monophyletic. Foltz et al., (2008) used a molecular clock calibrated with the crossover of *Leptasterias muelleri* through the Bering Strait as the prior probability. The estimated divergence between *L. hexactis* and *L. aequalis* using the putative mitochondrial control region and COI was 2.56 Mya and 3.08 Mya, respectively. Both estimates were averaged for the combined mtDNA divergence time and set as the normal distribution calibration point in this study. Starting trees were randomly generated and the tree prior assumed a Yule speciation process. For each BEAST analysis, the MCMC was performed for 10^7^ generations, sampling every 1,000 generations with a burn‐in of 10%. Summary statistics were generated and visualized in Tracer v1.6.0 (Drummond et al., 2012). Maximum clade credibility trees with median node heights of >50% posterior probabilities were calculated with TreeAnnotator v1.8.2 (Drummond et al., 2012) and were drawn in FigTree v1.4.0 (Rambaut, 2009). The analysis was run five times to confirm convergence and the combined results are reported.

### 2.7 Population analysis

D‐Loop and COI were analyzed both separately and as a single locus in all analyses below. The program DnaSP was used to calculate standard diversity indices including haplotype diversity (*h*) and nucleotide diversity (*π*_1_) for each population. DnaSP was used to calculate Tajima's *D*, Fu and Li's *D*, and Fu and Li's *F*. Neutrality statistics were considered significant when *p* < 0.05 and corrected for multiple comparisons. MEGA v5.2.2 (Tamura et al., 2011) was used to generate mean genetic distances between and within clades for all loci using the TN93 model for COI and D‐Loop and the HKY model for phased i51 haplotypes.

Arlequin v3.5 (Excoffier et al., 2005) was used to test for signatures of non‐neutral evolutionary forces on all loci by calculating Fu's *F*_S_ neutrality statistic (considered significant when *p* < 0.05). Arlequin was used to calculate fixation indices *F*_ST_ and Φ_ST_ (significant when *p* < 0.05). Φ_ST_ was calculated with the TN93 model of evolution for D‐Loop and COI and HKY was used for i51. Population structure was examined using an Analysis of Molecular Variance (AMOVA) across California populations grouped into three regions to test for previous structure found in Leptasterias populations around San Francisco Bay (Melroy et al., 2017) in additional sites and using more samples (Table 2): northern (populations north of Point Reyes), bay‐proximal (populations from Duxbury Reef to Mussel Rock), and southern (populations south of Half Moon Bay). Genetic differentiation was compared between (a) all three groupings (northern, southern, and bay‐proximal populations), and (b) northern and southern populations versus bay‐proximal populations. Mismatch distributions were generated in Arlequin and DnaSP for *L. aequalis* K and Clade Y samples. Other clades were omitted due to low sample sizes. Haplotype connections were exported from Arlequin and imported into HapStar v0.7 (Teacher & Griffiths, 2011) to build a minimum spanning network for all loci. Haplotype maps and haplotype network for i51 were constructed using indels as both informative and uninformative characters.

**TABLE 2:** Tamura‐Nei mean genetic distances for concatenated D‐Loop and COI mtDNA haplotypes

	*L. aequalis* A	*L. aequalis* K	*L. aequalis* D	*L. aequalis* B	Clade Y	Group 1	Clade Z	*L. hexactis*
*L. aequalis* A	0.001 ± 0.001							
*L. aequalis* K	0.027 ± 0.006	0.013 ± 0.002						
*L. aequalis* D	0.015 ± 0.004	0.028 ± 0.005	0.005 ± 0.002					
*L. aequalis* B	0.024 ± 0.005	0.009 ± 0.003	0.026 ± 0.005	0.009 ± 0.002				
Clade Y	0.013 ± 0.004	0.029 ± 0.006	0.018 ± 0.004	0.025 ± 0.005	0.006 ± 0.001			
Group 1	0.010 ± 0.003	0.030 ± 0.006	0.017 ± 0.004	0.027 ± 0.006	0.016 ± 0.004	0.005 ± 0.002		
Clade Z	0.018 ± 0.004	0.011 ± 0.003	0.020 ± 0.005	0.009 ± 0.003	0.020 ± 0.004	0.021 ± 0.005	0.012 ± 0.003	
*L. hexactis*	0.040 ± 0.007	0.042 ± 0.007	0.043 ± 0.007	0.040 ± 0.007	0.038 ± 0.007	0.043 ± 0.007	0.039 ± 0.007	0.004 ± 0.001
*L. camtschatica*	0.071 ± 0.009	0.072 ± 0.009	0.075 ± 0.009	0.070 ± 0.009	0.071 ± 0.009	0.069 ± 0.009	0.068 ± 0.009	0.052 ± 0.009

Note

Distances are between and within *Leptasterias* clades and species (± values are the standard error of the mean).

## 3 RESULTS

Historic samples amplified preferentially at COI but were often unsuccessful for amplification of D‐Loop or i51 (Table S2), therefore, historic population analysis uses only COI sequence data. Of 367 total COI sequences, 305 sea stars were collected between 2008 and 2014, and 61 sea stars were collected between 1897 and 1998 (Table 1). A 536 base‐pair region of COI showed 85 variable sites, 60 of which were parsimony‐informative. Seventy‐three haplotypes were identified, 34 of which were private haplotypes in contemporary samples and seven of which were private haplotypes found only in historic samples. A 297 base‐pair region of D‐Loop amplified in 322 individuals showed 48 variable sites, 40 of which were parsimony‐informative. There were 59 haplotypes of the amplified D‐Loop region and six indels within the region. Of the 59 total haplotypes, 45 were private. There were 55 variable sites within the 197 base‐pair intron region of i51, 15 of which were parsimony‐informative. There were a total of 46 i51 alleles and 27 of the alleles were private. Alleles consisted of 10 polymorphic sites and two indels. One indel consisted of a 13 base‐pair sequence repeat with alleles having between one and seven perfect repeats.

### 3.1 Phylogenetic analysis of contemporary and historical samples

**FIGURE 1:**
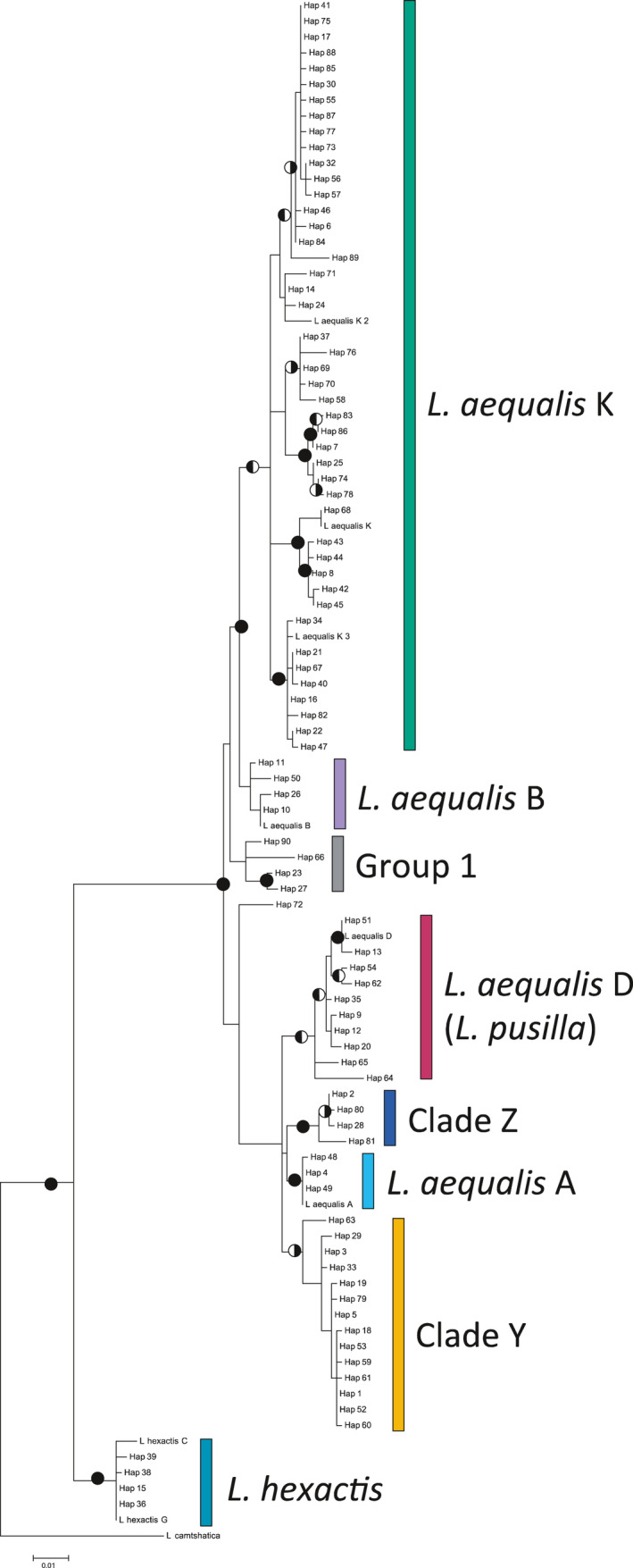
Maximum Likelihood tree for concatenated D‐Loop and COI mtDNA haplotypes of *Leptasterias*. Circles represent nodal support: right side shading represents bootstrap values of 70 or greater and left side shading represents Bayesian Posterior Probabilities of 95% or greater. Colors indicate groupings of haplotypes into clades. Reference sequences for *L. aequalis* clades D, K, A, and B and *L. hexactis* were obtained from GenBank

The first 378 bases of the COI locus were used to calculate base position changes for only COI (omitting N4DL and tRNA‐Arg); this region included 56 total variable sites: 14 first‐position changes, 4 s‐position changes, and 38 third‐position changes. When ML analyses were performed excluding third‐position changes for COI sequences overall topology was unchanged, though nodal support values increased slightly. Phylogenetic trees built with D‐Loop and COI separately had the same topology regardless of the method used to estimate the tree. Therefore, loci were concatenated for further phylogenetic analyses (Figure 1).

The concatenated haplotype phylogeny resolved six monophyletic clades with high statistical nodal support with the exception of four haplotypes, which are referred to as “Group 1” (Figure 1). The tree resolved all *L. aequalis* clades previously characterized by Foltz et al., (1996), Foltz et al., (2008): *L. aequalis* K, D, A, and B. We confirmed the presence of a monophyletic undescribed clade, Clade Y (Coleman et al., 2009; Melroy et al., 2017; Smith & Cohen, 2013), and uncovered an additional monophyletic undescribed clade, here termed Clade Z.

Genetic distances were calculated for concatenated D‐Loop and COI using the TN93 + G model (Table 2). Two main groups emerged from phylogenetic analysis: Grouping (A) *L. aequalis* B, *L. aequalis* K, and Group 1, and Grouping (B) *L. aequalis* D*, L. aequalis* A, Clade Y, and Clade Z. Genetic distances of clades within the two groupings ranged from 0.9%–2.0% within the first group and 1.3%–2.0% within Grouping B. Genetic distance ranged from 2.4%–2.9% between clades within the two groups. Intra‐clade genetic distances ranged from 0.10%–1.30%, with a mean genetic distance of 0.70%. *Leptasterias aequalis* K had the highest within‐clade genetic distance (1.30%). Mean inter‐clade genetic distance was higher (1.97%) with a range of 0.9%–2.9%.

**FIGURE 2:**
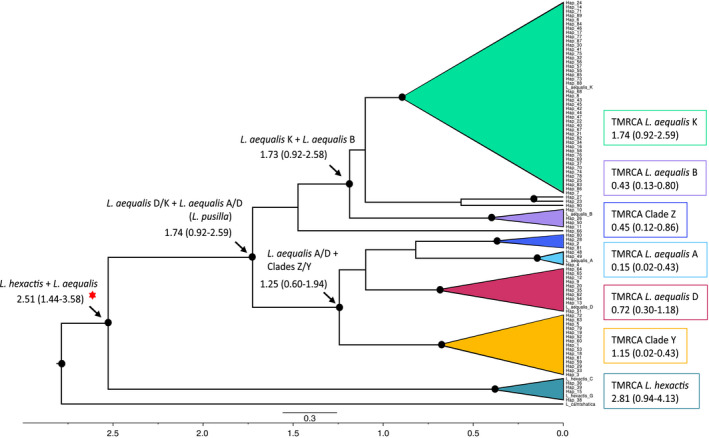
BEAST consensus tree for *Leptasterias* concatenated D‐Loop and COI mtDNA haplotypes constructed with Bayesian MCMC analysis (Drummond et al., 2012). *Leptasterias camtshatica* was included as the outgroup sister taxon. The *L. hexactis* and *L. aequalis* split, marked with a red asterisk, was estimated by Foltz et al., (2008) and used for calibration. Reference sequences were included from GenBank (see text for accession numbers). Black circles represent Bayesian Posterior Probability nodal support of 95% or greater. Estimates of divergence times are shown in millions of years and numbers in parentheses are 95% highest posterior density intervals. The scale bar shows the expected number of substitutions per site and the bottom grid axis represents time in millions of years with 0.0 as present day. Colors represent clades and are consistent with colors from Figure 1. Time to most recent common ancestor (TMRCA) is shown for each clade

Bayesian reconstruction in BEAST resulted in a tree with overall similar topology to that of Figure 1. The estimated mean rate of evolution for mtDNA was 0.0135 ± 0.0001 substitutions/site/My. Divergence times were estimated between clades and time to most recent common ancestor (TMRCA) was estimated within each clade (Figure 2). All effective sample sizes were over 1,000 with the exception of the split between *L. aequalis* A and Clade Z (*n*_ESS_ = 949), TMRCA for Clade Y (*n*_ESS_ = 517) and TMRCA for Clade Z (*n*_ESS_ = 987). The two main groupings of lineages (A and B) were estimated to have diverged between 1.74 and 1.25 Mya.

ML and Bayesian analysis of i51 alleles produced trees with the same topology. Samples obtained from Alaska populations, alleles CC, G, and SS, were used as outgroup sequences, as these samples were identified as *L. hexactis* through D‐Loop and COI barcoding. Alleles within *L. aequalis* were not phylogenetically resolved (Figure S1). The topology of the i51 phylogeny was unchanged when the 13 base‐pair repeat indel was used as informative. The i51 phylogenetic tree showed low resolution and did not further resolve the relationships within lineages of the putative *L. aequalis* complex.

### 3.2 Population genetic analysis

**FIGURE 3:**
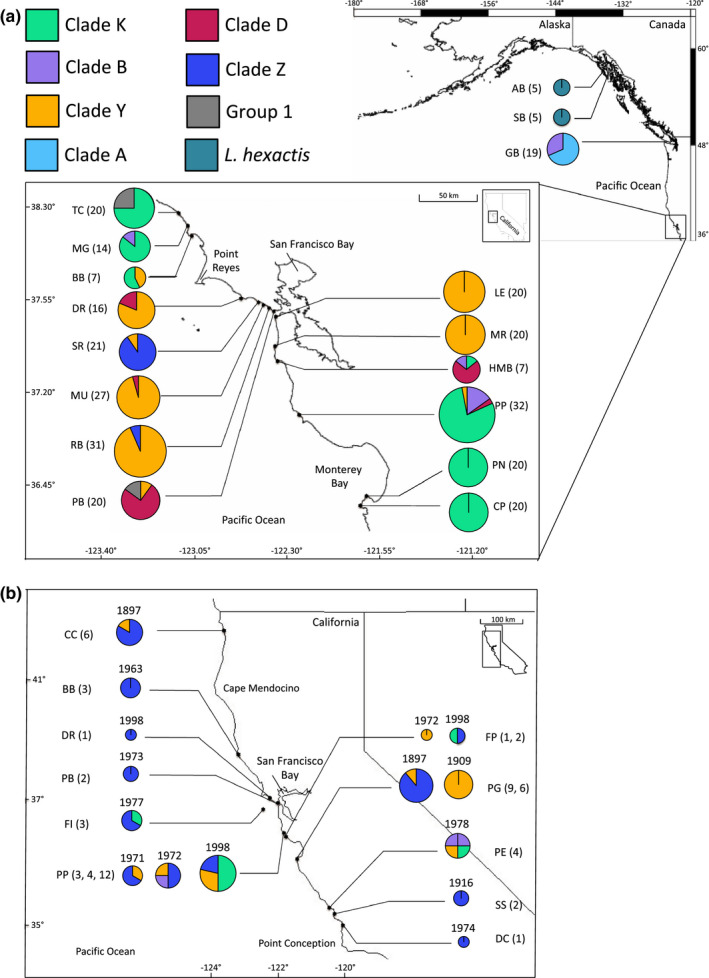
Clade frequencies of *Leptasterias* populations for (a) contemporary samples from central California to Alaska, and (b) historic samples collected in California. Colors represent clades and correspond to those found in Figure 1. Letters represent sample site, see Table 1, (*n* = sample size), numbers above the circles represent the collection year, and circle size is representative of sample size

Analysis of *Leptasterias* populations revealed 59 D‐Loop haplotypes and 46 i51 alleles across 17 sites and 73 COI haplotypes across 33 sampling sites and times. Mitochondrial haplotypes revealed a strong contemporary geographic pattern in which there were shared haplotypes proximal to the bay bracketed by distinct, shared haplotypes north and south of the bay (Figure S2). When mitochondrial haplotypes were delineated into clades, a genetic disconnect was confirmed with Clade Y found as bay‐associated, bracketed by distinct populations made up predominantly of *L. aequalis* K (Melroy et al., 2017; Smith & Cohen, 2013; Figure 3a). Clade Z was also found as bay‐proximal. Northern and southern populations were comprised of haplotypes that resolved into *L. aequalis* K and *L. aequalis* B. *Leptasterias aequalis* D was found in all regions. *Leptasterias aequalis* K and Clade Y were the most abundant clades found in the contemporary samples. Alaska population haplotypes were delineated as *L. hexactis*, and the Washington population comprised of haplotypes delineated as *L. aequalis* B and *L. aequalis* A. There were high numbers of private haplotypes for mtDNA and fewer private alleles for nuclear DNA (Table 1, Figure S2). The haplotype map for nuclear DNA revealed patterns in which high‐frequency haplotypes were abundant at central sites and present in almost all sites, revealing shared alleles between populations (Figure S2).

**FIGURE 4:**
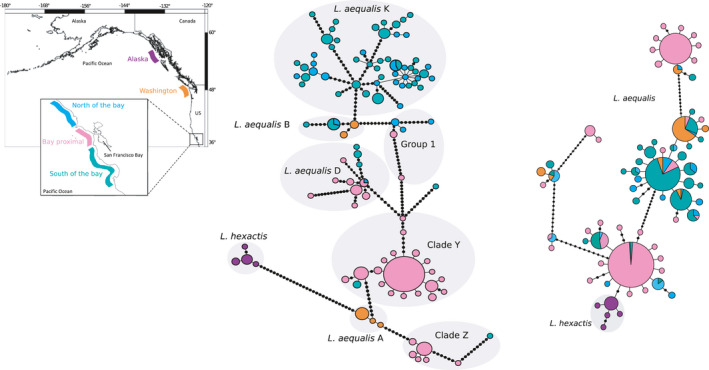
Haplotype network for concatenated D‐Loop and COI mtDNA haplotypes (middle) and nuclear i51 haplotypes with indels as informative characters (right). Circles represent haplotypes and circle size represents the frequency of haplotypes. Black circles represent missing haplotypes. Colors indicate population regions corresponding to the map (left). Grey shading represents clade delineation of haplotypes from phylogenetic analysis

The mtDNA minimum spanning network revealed many low‐frequency haplotypes separated by a large number of mutations (Figure 4). Haplotypes in Clade Y showed a more typical pattern of one high‐frequency haplotype with many low‐frequency haplotypes separated by one or two mutations. *Leptasterias aequalis* K was comprised of haplotypes found both north and south of San Francisco Bay, while Clade Y was comprised almost completely of haplotypes that were bay‐proximal. The i51 minimum spanning network (Figure 4) revealed four common alleles with other low‐frequency alleles separated by one or two mutations. Indels were included in the minimum spanning network and resulted in many mutations separating each cluster of alleles. When indels were excluded from the minimum spanning tree, alleles were separated by fewer mutations; however, a geographic pattern was still not evident (Figure S3).

Haplotype diversity for all populations ranged from 0.0 to 0.95 for mtDNA and 0.10 to 0.87 for i51 (Table 3). The lowest haplotype diversity values for both mitochondrial and nuclear loci were measured at sites with predominant Clade Y abundance, whereas, the highest haplotype diversities occurred at sites with high abundances of *L. aequalis* K. Nucleotide diversity at all sites ranged from 0.00 to 0.025 for mtDNA and 0.0004 to 0.010 for i51 (Table 3).

**TABLE 3:** Molecular diversity indices for *Leptasterias* spp. estimated for each population using concatenated D‐Loop and COI (mtDNA) and nuclear intron, i51, sequence data

Site	mtDNA						i51					
	Hd ± *SE*	*π* ± *SE*	Tajima's *D*	Fu's *F*_S_	Fu and Li's *D*	Fu and Li's *F*	Hd ± *SE*	*π* ± *SE*	Tajima's *D*	Fu's *F*_S_	Fu and Li's *D*	Fu and Li's *F*
AB	0.90 ± 0.16	0.0020 ± 0.001	−1.094	−1.405	−1.938	−1.113	0.02 ± 0.15	0.0008 ± 0.001	−1.112	−0.339	−1.243	−1.347
SB	0.40 ± 0.13	0.0005 ± 0.000	−0.817	0.090	−0.817	−0.772	0.87 ± 0.09	0.0105 ± 0.002	−0.432	3.379	−0.629	−0.652
GB	0.57 ± 0.11	0.0120 ± 0.002	1.722	7.562	1.337	**1.721**	0.56 ± 0.09	0.0039 ± 0.001	−0.235	11.130	1.044	0.774
TC	0.95 ± 0.03	0.0149 ± 0.015	0.871	−0.740	1.053	1.117	0.75 ± 0.04	0.0069 ± 0.017	−0.234	3.174	−0.456	−0.336
MG	0.85 ± 0.07	0.0102 ± 0.002	−0.158	4.137	0.145	0.082	0.80 ± 0.08	0.0063 ± 0.001	−0.515	0.158	−0.135	−0.263
BB	0.71 ± 0.13	0.0249 ± 0.004	2.055	4.527	1.454	**1.756**	0.74 ± 0.08	0.0062 ± 0.001	0.527	3.394	0.254	0.374
DR	0.33 ± 0.13	0.0072 ± 0.003	0.313	10.721	**1.544**	1.384	0.24 ± 0.20	0.0016 ± 0.001	**−1.602**	2.428	−2.024	−2.207
SR	0.49 ± 0.13	0.0048 ± 0.002	−0.852	2.321	1.292	0.720	0.05 ± 0.05	0.0004 ± 0.000	**−1.482**	4.058	−2.451	−2.514
MB	0.57 ± 0.12	0.0034 ± 0.002	**−2.142**	−0.560	**−2.770**	**−3.039**	0.31 ± 0.08	0.0023 ± 0.001	**−2.054**	0.956	−2.003	−2.396
RB	0.70 ± 0.06	0.0037 ± 0.002	−1.495	3.476	0.737	−0.006	0.13 ± 0.06	0.0008 ± 0.000	**−1.922**	0.995	**−3.902**	**−3.857**
PB	0.86 ± 0.06	0.0134 ± 0.003	0.060	2.141	1.090	0.912	0.55 ± 0.08	0.0054 ± 0.001	−1.346	9.261	−2.399	−2.424
LE	0.00	0.000	—	**−1.863**	—	—	0.28 ± 0.09	0.0014 ± 0.001	**−2.358**	0.990	**−3.751**	**−3.791**
MR	0.20 ± 0.12	0.0003 ± 0.000	**−1.513**	2.207	−2.053	−2.188	0.10 ± 0.07	0.0010 ± 0.001	**−1.883**	1.000	**−3.191**	**−3.259**
HMB	0.79 ± 0.15	0.0212 ± 0.005	0.215	3.039	0.134	0.137	0.82 ± 0.10	0.0087 ± 0.002	−0.677	0.843	1.281	−1.282
PP	0.90 ± 0.03	0.0175 ± 0.002	−0.748	1.502	−1.263	−1.228	0.87 ± 0.03	0.0079 ± 0.001	−0.673	−2.653	−0.387	−0.573
PN	0.78 ± 0.07	0.0112 ± 0.001	0.262	3.506	0.523	−0.075	0.61 ± 0.08	0.0046 ± 0.001	−1.179	−0.836	0.523	−0.006
CP	0.83 ± 0.06	0.0091 ± 0.001	0.317	0.851	0.250	−0.053	0.65 ± 0.08	0.0053 ± 0.001	−0.091	−1.022	0.250	0.173

Note

Bold values indicate significance (*p* < 0.05 for Tajima's *D*, Fu and Li's *D*, Fu and Li's *F*; *p* < 0.02 for Fu's Fs).

*Leptasterias* spp. pairwise comparisons (*F*_ST_ and Φ_ST_) for contemporary samples revealed significant population structure between most localities (Table S3). All but four mtDNA *F*_ST_ and Φ_ST_ values showed significant population differentiation for California sites. The only populations without significant differentiation were northern sites: Twin Cove and Marshall Gulch, and bay‐proximal sites: Rodeo Beach and Mussel Rock, Rodeo Beach and Lands End, and Lands End and Mussel Rock. Pairwise comparison values of mitochondrial and nuclear haplotypes between northern and southern sites were low, indicating genetic similarity. The AMOVA analysis for mtDNA and i51 reflected the genetic similarity between populations north of San Francisco Bay and south of San Francisco Bay. In both analyses, the predominant variation accounted for between‐group variation (Table S4) when northern and southern populations were grouped together and compared with central populations. High within‐population variation indicates the presence of sympatric clades.

**FIGURE 5:**
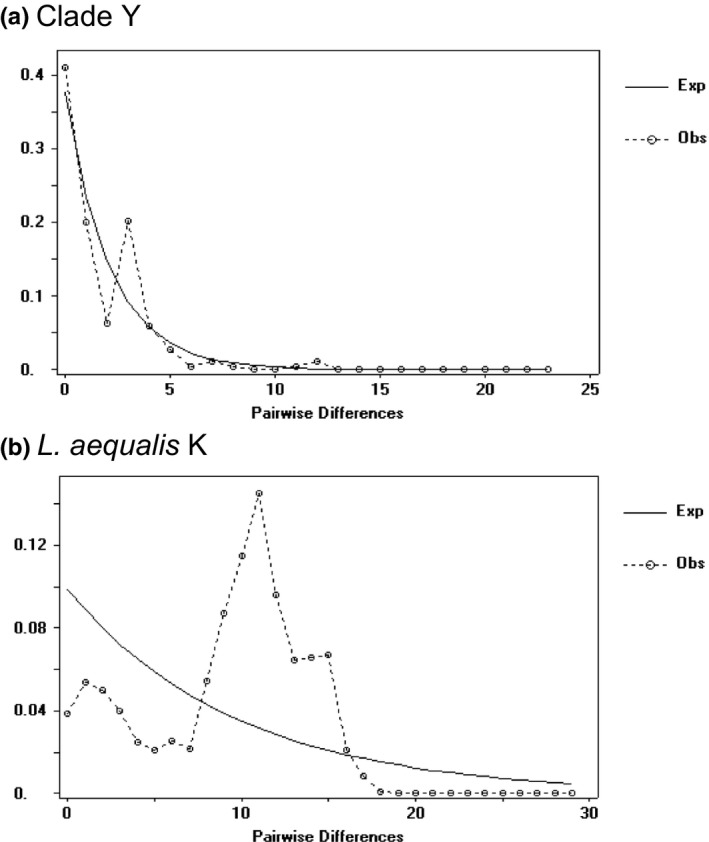
Mismatch distribution of pairwise distances among concatenated D‐Loop and COI mtDNA haplotypes for (a) *Leptasterias* spp. Clade Y haplotypes (Harpening's raggedness value *r* = 0, *p*‐value = 1), and (b) *L. aequalis* K haplotypes (Harpening's raggedness value *r* = 0.15, *p*‐value = 0.41) compared to expected frequencies (calculated in DnaSP v5.10 and Arlequin v3.5)

Significant negative Tajima's *D*, Fu's Fs, Fu and Li's Fs, and Fu, and Li's *D* statistics were calculated at Lands End, Muir Beach, and Mussel Rock across both mitochondrial and nuclear loci (Table 3). Mismatch distributions of mtDNA haplotypes for *L. aequalis* K and Clade Y did not differ significantly from the unimodal curves expected for a sudden demographic expansion or for a rapid spatial expansion (Figure 5). The raggedness index values were not significant for either clade and a hypothesis of sudden expansion could not be rejected (Table S5). Clade Y showed a steep peak in the distribution, consistent with a recent bottleneck or expansion event. The *L. aequalis* K distribution was slightly more ragged, but still consistent with a demographic expansion. While the unimodal distributions of both clades indicate recent population expansion, both expansion events and selective processes can result in a distribution of low diversity.

### 3.3 Historic samples

**FIGURE 6:**
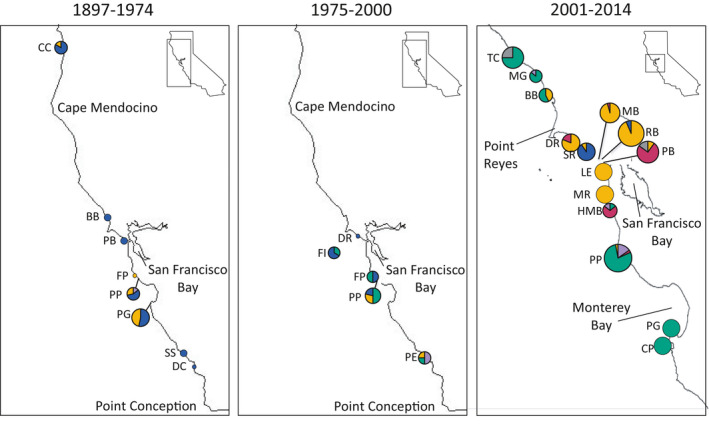
Frequency clade map for *Leptasterias* spp. between 1897 and 2014. Clades were delineated from COI haplotypes. Letters represent site code, size of circles represent sample size (see Table 1) and colors represent clade delineation (see Figure 1)

Historic samples successfully amplified at COI, however, successful amplification of D‐Loop or i51 was variable. COI haplotypes (from historic and contemporary samples) were used to build ML and Bayesian phylogenetic trees (not shown; the topology of supported branches did not differ from the concatenated mtDNA tree). Two dominant haplotypes resolved into Clades Z and Y, respectively, which were historically widespread (Figure 3), in contrast to contemporary samples, where these two dominant haplotypes were only found in bay‐proximal populations (with the exception of one individual at Pigeon Point). Historical sampling revealed two clades, Clades Z and Y, as more widespread and abundant than indicated by contemporary sampling. Historic samples showed Clade Z was once found across 800 km of coastline from Crescent City, CA to Diablo Canyon, CA and Clade Y was found across 750 km of coastline from Crescent City, CA to Piedras Blancas, CA (Figure 6). In contemporary samples, Clade Y was found at sites across 100 km of coastline with one individual found at Pigeon Point as the southern range limit. Clade Z was less abundant in contemporary samples, found only at Slide and Rodeo Beach, two sites separated by 8 km. Both clades were only found localized around San Francisco Bay in contemporary samples. One Clade Y individual (out of six total) was found in Pacific Grove in 1897. In 1909, all six individuals sampled in Pacific Grove were Clade Y. In 2014, zero Clade Y individuals were found in Pacific Grove. At Pigeon Point, one, one, and four individuals were found in 1971, 1972, and 1998, respectively. In 2014, one Clade Y individual was found at Pigeon Point with a more robust sampling scheme of 32 individuals. *Leptasterias aequalis* K was the most abundant clade at both Pigeon Point and Pacific Grove in contemporary samples.

## 4 DISCUSSION

### 4.1 Divergence times and phylogenetics

COI coupled with D‐Loop offered higher resolution of lineages within the *Leptasterias* genus than analysis using D‐Loop alone (Melroy et al., 2017) and revealed the presence of two potential species complexes historically grouped into one. Previously, *L. aequalis* D has been interchangeably referred to as *L. pusilla* (Foltz et al., 2008), implying the phylogenetic grouping found in this study of *L. aequalis* D, *L. aequalis* A, Clade Y, and Clade Z makes up a nominal species complex of *L. pusilla* that is separate from the *L. aequalis* complex. Genetic distances between the two potential species complexes (0.9%–2.9%, with a 1.97% mean genetic distance) are comparable to differences found in other divergent, brooding asteroid lineages (1.1%–4.3%, Hart et al., 2003). While genetic distances do not alone support the separation of clades into distinct species, additional loci and behavioral, morphological, or physiological analyses will help to resolve the relationship of lineages within the *Leptasterias* genus (e.g., Shaw & Cohen, 2015; Gong et al., 2019; Jaffe, 2020; Johnson et al., 2018; Johnson, 2020; Rupert, 2020).

The nuclear intron locus showed lower levels of variation than mitochondrial loci and did not resolve the putative *L. aequalis* into monophyletic lineages. Introns tend to accumulate mutations at a higher rate than exons and have a slower time to coalescence than mitochondrial loci (Hung et al., 2016), however, i51 is a short intron and might be experiencing genetic hitchhiking through selection on the exons. Hitchhiking would result in reduced observed variability compared with expected variability. The nuclear tree showed a starburst pattern, which could indicate recent speciation with shared ancestral polymorphisms or recurrent gene flow, however, it is challenging to draw conclusions using only this small locus.

Using mitochondrial data, divergence time between *Leptasterias* clades *L. pusilla* and *L. aequalis* was estimated to have occurred 1.74 Mya. TMRCA of *Leptasterias* spp. clades ranged from 0.15 to 1.74 Mya, suggestive of a recent species radiation event. Given these divergence times, *Leptasterias* clades were likely geographically and genetically isolated due to range fragmentation caused by glaciation events and sea‐level changes during the Pleistocene (Foltz et al., 2008). Following isolation in the Pleistocene, range expansion in the Holocene likely occurred, as seen in other taxa (Dawson et al., 2011; Ellingson & Krug, 2006; Hellberg et al., 2001; Jacobs et al., 2004; Marko, 1998). While Pleistocene glaciations may have driven speciation in *Leptasterias*, additional neutral and adaptive processes of divergence likely contributed to the maintenance of divergence and population structure.

### 4.2 Patterns of population structure

A previous study by Je Lee and Boulding (2009) found higher levels of temporal genetic stability for brooding littorinid gastropods than planktonic‐dispersing littorinids in British Columbia. However, this sea star study found seemingly low levels of temporal stability and high genetic turnover in a relatively short time period, 117 years, for low‐dispersing stars along the California coastline. Historic sampling revealed a widespread range of Clade Y and Clade Z, while contemporary sampling revealed restricted and localized ranges of both clades around the San Francisco Bay.

The dramatic shift in population structure for two clades over 117 years brings into question the processes that are maintaining divergence and driving genetic distribution of populations along the California coastline. The contemporary distribution of Clade Y around San Francisco Bay cannot be attributed to the formation of San Francisco Bay approximately 10,000 years ago (Atwater et al., 1977; Axelrod, 1981), as the speciation event of Clade Y predates the bay formation. While stochastic events such as variable hydrodynamic processes can affect low‐dispersing organisms, patterns of contemporary clade distribution might be more specifically explained by several mechanisms, including: (a) colonization events during the San Francisco bay formation, (b) ocean circulation and transport processes, (c) local adaptation of Clade Y to bay effluent conditions, or (d) competitive success of *L. aequalis* K.

#### 4.2.1 Colonization events during the San Francisco bay formation

The historically widespread and abundant Clade Y or Clade Z haplotypes could represent a source of founders colonizing the bay area. It is possible Clade Y individuals inhabited the area that eventually formed San Francisco Bay and colonized coastal areas around the bay following formation, resulting in their current localized distribution. Colonization events are supported by negative neutrality statistics and low haplotype diversities at Clade Y sites, as seen in other taxa experiencing genetic bottlenecks and reduced diversity associated with colonization (Marko, 1998; Hess et al., 2011) Concurrently, the unimodal mismatch curve for Clade Y suggested a recent population expansion and could reflect expansion around San Francisco Bay.

#### 4.2.2 Ocean circulation and transport processes

*Leptasterias* lack a planktonic dispersal stage, however, they can be considered epi‐planktonic through long‐distance dispersal on algal rafts (Highsmith, 1985). While these events are likely infrequent, when they do occur, rafters could be affected by ocean circulation processes much as planktonic dispersers are. The central CA region of coastline in this study has two potential physical barriers to dispersal for rafting organisms: San Francisco Bay and Point Reyes.

Estuarine outflow from San Francisco Bay is a potential barrier to dispersal due to uninhabitable effluent conditions. Young or adults on rafts might experience mortality due to conditions associated with San Francisco Bay effluent: low salinity, high temperatures, pollutants from wastewater run‐off (Conomos et al., 1985; Luoma & Cloern, 1982; Nichols et al., 1986), or even facilitated offshore transport. Puritz and Toonen (2011) found reduced genetic diversity and connectivity in the planktonic disperser *Patiria miniata* across areas of high human impact and pollutant run‐off in the Southern California Bight attributed to larval mortality. San Francisco Bay effluent could act as a similar barrier to dispersal in *Leptasterias*, though additional genetic assays of other intertidal organisms around the bay outflow would help elucidate this theory.

The Point Reyes peninsula is a prominent geographic feature in the range of *Leptasterias*. Several studies indicate Point Reyes is a barrier to dispersal for other taxa: in the low‐dispersing genera*, Alderia* (Ellingson & Krug, 2006), *Tigriopus* (Edmands, 2001), and *Nucella* (Marko, 1998), and in the high dispersing species, *Mesocentrotus franciscanus* (Moberg & Burton, 2000). Retention embayments occur both north and south of Point Reyes, which can retain nearshore waters and entrain non‐local propagules (Morgan et al., 2009; Wing et al., 1998). These retention zones could effectively limit connectivity between southern and bay‐proximal *Leptasterias* populations and populations north of Point Reyes.

The Monterey Bay region also has documented retention zones (Graham & Largier, 1997; Vander Woude et al., 2006) which could facilitate connectivity between northern populations and southern populations. The California current is southward driven during upwelling months (Checkley & Barth, 2009; Huyer, 1983; Largier et al., 1993) and water entrained in the Point Reyes eddy will eventually move offshore or south to Monterey (Rosenfeld et al., 1994; Steger et al., 2000). Oceanic current conditions along the coastline provide a potential for water transport north to south, which could connect northern and southern populations while reducing movement of water toward the San Francisco Bay gateway.

#### 4.2.3 Local adaptation of Clade Y to bay effluent conditions

Rather than divergence due to neutral processes, *Leptasterias* divergence could be the result of adaptive processes. Interestingly, *Leptasterias* patterns of clade distributions appear to coincide with regions of upwelling exposure in central California. Clade Y might be locally adapted to warm, low salinity conditions from San Francisco effluent affecting local coastal areas (Melroy et al., 2017; Smith & Cohen, 2013).

Intense upwelling zones span from Point Arena to Cape Mendocino (Bakun, 1990; Huyer & Kosro, 1987), and occur near Año Nuevo (Rosenfeld et al., 1994). *Leptasterias aequalis* K and *L. aequalis* B occur at upwelling exposed regions north of Point Reyes and south of Half‐Moon Bay (Figure 6). Bay‐proximal populations are exposed to the warm, low salinity effluent from San Francisco Bay. Low haplotype diversities and negative neutrality statistics at the mitochondrial and nuclear loci used in this study could reflect selection upon other genes favoring Clade Y individuals at bay‐proximal sites. Adaptive divergence is consistent with expectations of brooders that lack a highly dispersive life stage (reviewed by Sanford & Kelly, 2011; Sotka, 2012; Strathmann, 1986). While the genetic break of *Leptasterias* clades around the bay area appears to be upwelling associated, other factors associated with estuarine effluent may be causing further differentiation of Clade Y. Behavioral assays assessing the tolerance of clades to variable temperature and salinity conditions are an area of ongoing investigation (Contreras & Cohen, 2014; Shaw & Cohen, 2015, Braun et al., 2016; Rupert, 2020), though are made difficult due to population declines from SSWD, discussed below.

#### 4.2.4 Competitive success of *L. aequalis* K

Competition between clades could be another viable hypothesis for the distribution of *Leptasterias* clades in the central California region. *Leptasterias aequalis* K could outcompete Clade Y except at bay‐proximal sites where Clade Y is locally adapted. While historic sample sizes are low, the presence of *L. aequalis* K was detected in only four populations between 1977 and 1998 (and not detected in any before 1977). In comparison, *L. aequalis* K was the second most abundant clade in contemporary samples. It is possible that *L. aequalis* K has increased in abundance at sites that are not bay‐proximal, but were once presumably dominated by Clades Z or Y. Low sample sizes of historic collections make this hypothesis difficult to test, though physiological assays could reveal differences in clade tolerances to local conditions.

### 4.3 Conclusions

Pleistocene dated divergence times for *Leptasterias* clades suggest glacial cycles contributed to reproductive isolation. Phylogenetic analysis and genetic distances indicate the presence of two distinct species complexes. Historic genetic sampling revealed Clades Y and Z as previously widespread and abundant along the California coastline, while contemporary sampling revealed these clades as bay‐localized. Both selection and demographic events can result in low haplotype diversities, negative neutrality statistics, and unimodal mismatch distributions; the maintenance of divergence between *Leptasterias* clades might be due to both neutral and adaptive processes of divergence.

Findings by Je Lee and Boulding (2009) led us to predict high temporal stability for the low‐dispersing *Leptasterias,* largely attributed to low juvenile mortality between generations. Instead, high levels of population structure on a spatial scale (over 1,500 km of Pacific coastline) and high genetic variability on a temporal scale (117 years) were observed. Another study also reported high temporal variability for brooding lineages of *Pygospio elegans*, indicating patterns may vary across taxa and across time periods (Kesäniemi et al., 2014). There are likely several factors contributing to the contradictions of our temporal prediction including stochastic processes affecting rafting organisms and mass mortality events. Low‐dispersers have low effective population sizes and are vulnerable to extinction and colonization events. These types of demographic events could cause high temporal variability depending upon the timescale of each.

Mass mortality events have the ability to dramatically alter the distribution and composition of clades at local sites, especially for brooding organisms. There are many examples of population density variation over short timescales in echinoderms through mass die‐offs and sharp population increases (Uthicke et al., 2009). Indeed, in just the timescale of sample collection for this study between 2008 and 2014, several such die‐offs of *Leptasterias* were observed at local sites. In 2010–11, *Leptasterias* disappeared from Mussel Rock, a sampling site where they were previously abundant, and where they have since not been found as of October 2020 (Jaffe et al., 2019; pers. obs., M. Duncan, M. Kelley, B. Huey). A harmful algal bloom was the attributed cause of mortality for *Leptasterias* populations along the Sonoma coast in 2011 (Jurgens et al., 2015). Beginning in 2013, SSWD, putatively attributed to a densovirus, caused massive population declines in *Leptasterias* and many other sea star genera (Harvell et al., 2019; Hewson et al., 2014, but see Hewson et al., 2018; Hewson et al., 2019; MARINe, 2015; Miner et al., 2018; Menge et al., 2016; Jaffe et al., 2019) and allele frequency shifts in *Pisaster ochraceus* (Schiebelhut et al., 2018). The frequency of these documented events over 10 years suggests local extinction events might have been common in the evolutionary history of *Leptasterias* as they have been for other echinoderms (Uthicke et al., 2009). The decline in abundance and range of Clades Y and Z illustrate brooding species’ susceptibility to variation in population density. This study provides a record of population structure in *Leptasterias* sea stars over 117 years along the California coastline and can be used to understand changing population dynamics caused by large‐scale mortality events.

Range shifts of native species have the potential to heavily impact community structure and function in regions experiencing expansions (Sorte et al., 2010) and poleward range shifts have been documented in many invertebrate species (Lonhart et al., 2019; Sanford et al., 2019; Sorte et al., 2010). We cannot rule out climate change as a potential factor in the range shifts found in this study. *Leptasterias* function as important predators in the intertidal environment by preying upon snails, limpets, barnacles, and juvenile mussels. Changes in population abundance of *Leptasterias* could impact standing algal stocks by indirectly affecting grazing by herbivores (Gravem & Morgan, 2019). Long‐term changes in upwelling processes associated with climate change such as stronger upwelling‐favorable winds, colder water, and a higher frequency of upwelling occurrences (García‐Reyes & Largier, 2010) have the potential to impact selective and demographic forces that can lead to further shifts in population dynamics.

Historic genetic sampling has important implications in conservation management practices through monitoring population genetic diversity and interpreting environmental influences on diversity (Fenderson et al., 2020; Nielsen & Hansen, 2008). We found dramatic change in a genus of sea stars over a relatively short time span on the California coastline and we suggest several mechanisms for how the environmental landscape has shaped the recent evolutionary history of a low‐dispersing sea star. We recommend further studies to understand the species delineation within this genus through morphological and physiological analysis. Additional monitoring of genetic diversity over time following sea star wasting disease, paired with this dataset, would be a valuable look at changing genetic diversity caused by mass mortality events.

## CONFLICT OF INTEREST

The authors have no conflicts of interest to declare.

## AUTHOR CONTRIBUTIONS

**Laura Melroy:** Data curation (lead); formal analysis (lead); investigation (equal); methodology (equal); software (lead); visualization (lead); writing – original draft (lead); writing – review and editing (equal). **Cynthia Sarah Cohen:** Conceptualization (lead); data curation (supporting); funding acquisition (lead); investigation (equal); project administration (lead); resources (lead); supervision (lead); writing – review and editing (equal).

## Supporting information

Supplementary Material

Supplementary Material

## Data Availability

*Leptasterias* spp. haplotype GenBank identification numbers can be found in Supplementary File 2. *Leptasterias* sample information, including IDs for museum samples, is found in Table 1. Table S2 shows all museum samples obtained, site of collection, and success of genotyping at each locus. https://doi.org/10.5061/dryad.4mw6m907m
