# Supplementary material for: Temporal and spatial variation in population structure among brooding sea stars in the genus Leptasterias
Source: Ecol Evol. 2021 Mar 4;11(7):3313–31. doi: 10.1002/ece3.7283 (PMC8019026; doi:10.1002/ece3.7283)
Supplement: Supplementary file 1 — Supplementary Material [file ECE3-11-3313-s001.docx]

**Supplementary Files**

**Table S1.** GenBank accession numbers for reference sequences used in phylogenetic analysis.

| *Leptasterias* clade | COI Accession Number | D-Loop Accession Number |
| --- | --- | --- |
| *L.* *aequalis* A | AF162089 | AF162111 |
| *L.* *aequalis* B | AF162090 | U37563 |
| *L.* *aequalis* D | AF162092 | AF162112 |
| *L.* *aequalis* K | AF162109 | AF162128 |
| *L.* *aequalis* K | AF162099 | AF162118 |
| *L.* *aequalis* K | AF162100 | AF162119 |
| *L. hexactis* C | AF162091 | U37564 |
| *L. hexactis* G | AF162095 | AF162115 |
| *L. camtschatica* | AF162104 | AF162123 |

**Table S2.** **Summary of success in genotyping historic samples at each locus.** Samples from Lonesome Cove, Franklin Point, and Pigeon Point collected in 1998 were obtained from Foltz, LSU). All other samples were obtained from the California and Academy of Sciences and ID numbers indicate the CASIZ identifier from their Invertebrate Zoology Collection. Letters indicate different specimens stored in the same collection jar, under the same identifying number. Sample sites are listed north to south.

| *Site* | Collection year | Total samples collected | Specimen ID | 16S genotyped | I51genotyped | COI genotyped |
| --- | --- | --- | --- | --- | --- | --- |
| Lonesome Cove, WA | 1998 | 2 | Q69, Q71 | 1 | 0 | 1 |
| Crescent City, CA | 1897 | 7 | 191756A-G | 0 | 7 | 0 |
| Point Arena, CA | 1961 | 1 | 115846 | 0 | 0 | 0 |
| Horseshoe Cove | 1961 | 2 | 201224A-B | 0 | 0 | 0 |
| Bodega Head, CA | 1962 | 1 | 201226 | 0 | 0 | 0 |
| Bodega Head, CA | 1963 | 3 | 201225, 115521A-B | 0 | 2 | 3 |
| Duxbury Reef, CA | 1998 | 1 | 115493 | 0 | 1 | 1 |
| Point Bonita, CA | 1973 | 2 | 115524A-B | 0 | 2 | 2 |
| SE Farallons, CA | 1974 | 1 | 115498 | 0 | 0 | 0 |
| SE Farallons, CA | 1977 | 3 | 4826A-C | 3 | 3 | 3 |
| Pigeon Point, CA | 1971 | 3 | 7676A-C | 1 | 3 | 3 |
| Pigeon Point, CA | 1972 | 8 | 7642A-D, 191755A-D | 5 | 8 | 4 |
| Pigeon Point, CA | 1998 | 20 | K12-32 | 11 | 0 | 12 |
| Franklin Point, CA | 1972 | 1 | 7645 | 0 | 0 | 1 |
| Franklin Point, CA | 1998 | 4 | J96-99 | 0 | 0 | 2 |
| Pacific Grove, CA | 1897 | 11 | 191756AH-R | 0 | 11 | 8 |
| Pacific Grove, CA | 1909 | 8 | 108854A-H | 0 | 0 | 0 |
| Pacific Grove, CA | 1966 | 1 | 116023 | 0 | 0 | 0 |
| Piedras Blancas, CA | 1975 | 1 | 115490 | 0 | 0 | 0 |
| Pedras Blancas,CA | 1978 | 4 | 164031, 115497A-B, 108853 | 0 | 0 | 4 |
| San Simeon, CA | 1916 | 2 | 115491A-B | 0 | 1 | 2 |
| Diablo Canyon, CA | 1974 | 2 | 135003 | 0 | 1 | 1 |
| Diablo Canyon, CA | 1975 | 1 | 158190 | 0 | 0 | 0 |
| Diablo Canyon, CA | 1977 | 1 | 164046 | 0 | 0 | 0 |

**Figure S1.** Bayesian tree for i51 haplotypes using indels coded as simple characters (Simmons and Ochoterena 2000). Numbers at the nodes indicate Bayesian Posterior Probabilities. Haplotypes CC, G, and SS belong to individuals identified as *L. hexactis* using mtDNA haplotypes and were used to root the tree.

**Figure S2.** Haplotype frequency maps for contemporary *Leptasterias* samples. Haplotype maps show frequency of haplotypes in populations for mtDNA (top) and nuclear i51 (bottom). Letters indicate the population code (n=sample size). Colors represent different haplotypes, white wedges represent private haplotypes and numbers within and next to the white represent number of private haplotypes within the population.

**Figure S3.** Haplotype network for i51 haplotypes excluding indels. Region key found left. Circles represent haplotypes and size of circles represents the frequency of haplotypes. Black circles represent missing haplotypes. Colors represent population regions. Grey shading represents clade delineation of haplotypes.

**Table S3.** Pairwise comparison of genetic structure for *L. hexactis* and *L. aequalis* populations. Values for i51 are shown above the diagonal and values for mtDNA are shown below the diagonal for a) Φ_ST_ and b) F_ST_. Bold numbers indicate significant values with p < 0.05. See Table 1 for location abbreviations.

a)

|  | AB | SB | GB | TC | MG | BB | DR | SR | RB | MB | PB | LE | MR | HMB | PP | PN | CP |
| --- | --- | --- | --- | --- | --- | --- | --- | --- | --- | --- | --- | --- | --- | --- | --- | --- | --- |
| AB | - | **0.190** | **0.730** | **0.673** | **0.792** | **0.724** | **0.860** | **0.946** | **0.812** | **0.917** | **0.676** | **0.868** | **0.900** | **0.636** | **0.647** | **0.804** | **0.764** |
| SB | -0.087 | - | **0.386** | **0.436** | **0.523** | **0.382** | **0.488** | **0.615** | **0.496** | **0.636** | **0.362** | **0.530** | **0.543** | **0.323** | **0.468** | **0.631** | **0.559** |
| GB | **0.780** | **0.786** | - | **0.236** | **0.496** | **0.194** | **0.033** | **0.071** | **0.015** | **0.101** | **-0.017** | **0.137** | **0.081** | **0.098** | **0.308** | **0.530** | **0.460** |
| TC | **0.745** | **0.753** | **0.495** | - | **0.153** | 0.002 | **0.386** | **0.442** | **0.389** | **0.494** | **0.189** | **0.441** | **0.422** | **0.206** | **0.070** | **0.162** | **0.108** |
| MG | **0.836** | **0.844** | **0.602** | 0.068 | - | **0.140** | **0.684** | **0.782** | **0.664** | **0.796** | **0.412** | **0.722** | **0.736** | **0.349** | 0.008 | **0.082** | 0.071 |
| BB | **0.669** | **0.683** | **0.327** | **0.133** | **0.230** | - | **0.408** | **0.523** | **0.378** | **0.549** | **0.106** | **0.446** | **0.435** | **0.147** | 0.044 | **0.184** | **0.099** |
| DR | **0.851** | **0.859** | **0.478** | **0.662** | **0.767** | **0.430** | - | 0.022 | -0.011 | 0.001 | **0.046** | **0.043** | -0.097 | **0.216** | **0.427** | **0.675** | **0.612** |
| SR | **0.907** | **0.913** | **0.548** | **0.729** | **0.816** | **0.657** | **0.667** | - | 0.001 | **0.022** | **0.085** | **0.135** | -0.027 | **0.357** | **0.461** | **0.733** | **0.678** |
| RB | **0.925** | **0.931** | **0.658** | **0.758** | **0.849** | **0.624** | **0.365** | **0.802** | - | 0.011 | **0.049** | **0.047** | -0.038 | **0.254** | **0.436** | **0.665** | **0.604** |
| MB | **0.918** | **0.922** | **0.659** | **0.770** | **0.853** | **0.640** | **0.324** | **0.781** | **0.075** | - | **0.129** | **0.059** | -0.052 | **0.408** | **0.513** | **0.760** | **0.707** |
| PB | **0.770** | **0.776** | **0.433** | **0.576** | **0.659** | **0.433** | **0.455** | **0.609** | **0.636** | **0.653** | - | **0.130** | **0.095** | **0.082** | **0.269** | **0.466** | **0.384** |
| LE | **0.992** | **0.998** | **0.733** | **0.797** | **0.895** | **0.714** | **0.543** | **0.881** | 0.024 | **0.144** | **0.706** | - | -0.006 | **0.339** | **0.477** | **0.713** | **0.640** |
| MR | **0.987** | **0.993** | **0.729** | **0.795** | **0.892** | **0.708** | **0.534** | **0.876** | 0.024 | **0.140** | **0.703** | -0.001 | - | **0.322** | **0.462** | **0.716** | **0.637** |
| HMB | **0.722** | **0.732** | **0.373** | **0.450** | **0.549** | **0.286** | **0.502** | **0.606** | **0.705** | **0.725** | **0.168** | **0.785** | **0.780** | - | **0.260** | **0.447** | **0.376** |
| PP | **0.742** | **0.747** | **0.506** | **0.061** | **0.096** | **0.207** | **0.659** | **0.709** | **0.739** | **0.751** | **0.571** | **0.767** | **0.765** | **0.443** | - | **0.057** | **0.043** |
| PN | **0.813** | **0.820** | **0.606** | **0.074** | **0.105** | **0.261** | **0.749** | **0.801** | **0.824** | **0.830** | **0.655** | **0.861** | **0.859** | **0.552** | **0.076** | - | 0.031 |
| CP | **0.841** | **0.848** | **0.622** | **0.230** | **0.325** | **0.354** | **0.768** | **0.818** | **0.843** | **0.847** | **0.675** | **0.884** | **0.881** | **0.572** | **0.240** | **0.243** | - |

b)

|  | AB | SB | GB | TC | MG | BB | DR | SR | RB | MB | PB | LE | MR | HMB | PP | PN | CP |
| --- | --- | --- | --- | --- | --- | --- | --- | --- | --- | --- | --- | --- | --- | --- | --- | --- | --- |
| AB | - | **0.269** | **0.535** | **0.422** | **0.473** | **0.415** | **0.701** | **0.847** | **0.543** | **0.555** | **0.400** | **0.549** | **0.628** | **0.450** | **0.353** | **0.456** | **0.503** |
| SB | -0.016 | - | **0.297** | **0.160** | **0.158** | **0.119** | **0.475** | **0.660** | **0.336** | **0.343** | **0.162** | **0.323** | **0.407** | **0.155** | **0.118** | **0.206** | **0.258** |
| GB | **0.269** | **0.438** | - | **0.263** | **0.234** | **0.202** | **0.511** | **0.642** | **0.421** | **0.430** | **0.263** | **0.421** | **0.456** | **0.240** | **0.152** | **0.271** | **0.273** |
| TC | **0.072** | **0.250** | **0.206** | - | **0.081** | 0.037 | **0.428** | **0.541** | **0.342** | **0.335** | **0.149** | **0.329** | **0.387** | **0.154** | **0.039** | 0.017 | **0.062** |
| MG | **0.132** | **0.319** | **0.264** | **0.101** | - | 0.033 | **0.436** | **0.616** | **0.311** | **0.306** | **0.112** | **0.329** | **0.413** | **0.156** | 0.005 | **0.079** | **0.081** |
| BB | 0.123 | **0.346** | **0.275** | **0.079** | **0.149** | - | **0.396** | **0.563** | **0.284** | **0.278** | **0.089** | **0.295** | **0.373** | **0.123** | 0.017 | 0.040 | 0.037 |
| DR | **0.490** | **0.654** | **0.511** | **0.348** | **0.424** | **0.475** | - | 0.021 | **0.095** | 0.027 | **0.100** | **0.477** | **0.603** | **0.443** | **0.334** | **0.459** | **0.428** |
| SR | **0.286** | **0.451** | **0.374** | **0.220** | **0.279** | **0.292** | **0.519** | - | **0.161** | **0.086** | **0.309** | **0.588** | **0.708** | **0.623** | **0.434** | **0.573** | **0.541** |
| RB | **0.278** | **0.441** | **0.367** | **0.215** | **0.273** | **0.284** | **0.507** | **0.378** | - | 0.011 | **0.065** | **0.233** | **0.366** | **0.343** | **0.267** | **0.368** | **0.342** |
| MB | **0.230** | **0.389** | **0.328** | **0.183** | **0.235** | **0.240** | **0.455** | **0.326** | **0.082** | - | **0.087** | **0.322** | **0.450** | **0.338** | **0.262** | **0.362** | **0.335** |
| PB | **0.122** | **0.298** | **0.249** | **0.095** | **0.145** | **0.139** | **0.391** | **0.262** | **0.233** | **0.205** | - | **0.123** | **0.217** | **0.144** | **0.100** | **0.166** | **0.149** |
| LE | **0.615** | **0.757** | **0.588** | **0.429** | **0.514** | **0.589** | **0.746** | **0.592** | **0.084** | **0.218** | **0.446** | - | 0.026 | **0.331** | **0.265** | **0.357** | **0.329** |
| MR | **0.473** | **0.627** | **0.502** | **0.345** | **0.417** | **0.462** | **0.654** | **0.509** | 0.131 | **0.154** | **0.361** | 0.004 | - | **0.399** | **0.314** | **0.416** | **0.387** |
| HMB | 0.104 | **0.318** | **0.256** | **0.077** | **0.133** | **0.125** | **0.448** | **0.273** | **0.266** | **0.223** | **0.124** | **0.558** | **0.437** | - | **0.096** | **0.200** | **0.236** |
| PP | **0.076** | **0.244** | **0.200** | **0.045** | **0.084** | **0.079** | **0.325** | **0.213** | **0.208** | **0.179** | **0.093** | **0.391** | **0.321** | **0.059** | - | **0.043** | **0.039** |
| PN | **0.159** | **0.332** | **0.278** | **0.124** | **0.175** | **0.173** | **0.421** | **0.291** | **0.285** | **0.250** | **0.166** | **0.500** | **0.416** | **0.157** | **0.124** | - | 0.017 |
| CP | **0.122** | **0.298** | **0.249** | **0.095** | **0.145** | **0.139** | **0.391** | **0.262** | **0.257** | **0.233** | **0.137** | **0.471** | **0.387** | **0.124** | **0.094** | **0.159** | - |

**Table S4.** AMOVA results comparing populations grouped geographically with northern and southern populations either combined (a) or separate (b). See Table 1 for detailed regional groupings. Permutations (16,000) were carried out for each AMOVA.

|  | Source of variation | Df | Sum of square | Variance  components | Percentage  of variation | Fixation  Indices | P-value |
| --- | --- | --- | --- | --- | --- | --- | --- |
| *a) Two regional groupings: northern and southern vs. bay-proximal* | | | | | | | |
| mtDNA | Among groups | 1 | 1238.536 | 8.713 | 58.08 | F_CT_=0.581 | <0.00001 |
|  | Among populations within groups | 12 | 663.460 | 2.706 | 18.04 | F_SC_=0.430 | <0.00001 |
|  | Within populations | 259 | 927.627 | 3.582 | 23.88 | F_ST_=0.761 | <0.00001 |
|  | Total | 272 | 2829.623 | 15.001 |  |  |  |
|  |  |  |  |  |  |  |  |
| i51 | Among groups | 1 | 655.518 | 2.279 | 27.42 | F_CT_=0.274 | <0.00001 |
|  | Among populations within groups | 12 | 764.783 | 1.620 | 19.49 | F_SC_=0.268 | <0.00001 |
|  | Within populations | 512 | 2259.698 | 4.413 | 53.1 | F_ST_=0.469 | 0.00293 |
|  | Total | 525 | 3679.998 | 8.312 |  |  |  |
|  |  |  |  |  |  |  |  |
| *b) Three regional groupings: northern vs. southern vs. bay proximal* | | | | | | | |
| mtDNA | Among groups | 2 | 1265.136 | 7.182 | 52.89 | F_SC_=0.441 | <0.00001 |
|  | Among populations within groups | 11 | 634.173 | 2.823 | 20.78 | F_SC_=0.737 | <0.00001 |
|  | Within populations | 259 | 926.137 | 3.576 | 26.33 | F_ST_=0.529 | <0.00001 |
|  | Total | 272 | 2825.447 | 13.581 |  |  |  |
|  |  |  |  |  |  |  |  |
| i51 | Among groups | 2 | 30.724 | 0.084 | 18.10 | F_CT_=0.181 | <0.00001 |
|  | Among populations within groups | 11 | 31.971 | 0.071 | 15.22 | F_SC_=0.186 | <0.00001 |
|  | Within populations | 512 | 158.199 | 0.309 | 66.68 | F_ST_=0.333 | 0.00196 |
|  | Total | 525 | 220.894 | 0.463 |  |  |  |

**Table S5.** Parameter values for the sudden and spatial expansion models for the mismatch distribution of two *Leptasterias* clades.

|  | Sudden Expansion Model | | | | | |
| --- | --- | --- | --- | --- | --- | --- |
|  | Θ_0_ | Θ_1_ | τ | Goodness-of-fit  P-value | Harpending’s Raggedness | P-value |
| *L. aequalis* K | 0 | 37.72 | 13.82 | 0.15 | 0.01 | 0.41 |
| Clade Y | 0 | 99999 | 0 | 0 | 0.06 | 1 |
|  |  |  |  |  |  |  |
|  | Spatial Expansion Model | | | | | |
|  | Θ | M | τ | Goodness-of-fit  P-value | Harpending’s Raggedness | P-value |
| *L. aequalis* K | 1.63 | 18.23 | 11.96 | 0.08 | 0.01 | 0.94 |
| Clade Y | 1.76 | 0.07 | 11.28 | 0.74 | 0.06 | 0.83 |
